# Supplementary material for: A Study on the Planarian Model Confirms the Antioxidant Properties of Tameron against X-ray- and Menadione-Induced Oxidative Stress
Source: Antioxidants (Basel). 2023 Apr 18;12(4):953. doi: 10.3390/antiox12040953 (PMC10136237; doi:10.3390/antiox12040953)
Supplement: Supplementary file 1 [file antioxidants-12-00953-s001.zip › antioxidants-2309994-supplementary.pdf]

## Supplementary Materials

**Table S1.** Selected gene groups for RT-PCR analysis of neoblast marker genes.

| Gene name | Description | NCBI Reference Sequence | Primer F | Primer R |
|---|---|---|---|---|
| *smed-hnf-4* | olfactory receptor 4C6 [Homo sapiens] | JF802199.1 | CGACGCGTTAGACGCATAGAC | GCTTCCTCGATGCAATATCCAT |
| *Smed-gata456* | transcription factor GATA-6 [Homo sapiens] | JF802198.1 | TGTGTGAACTGTGGAGCTAGCAA | TTGAGAGAACCTGTCGCATTCAT |
| *Smed-prox-1* | prospero homeobox protein 1 [Homo sapiens] | JQ425149.1 | TCATTTACGAAAGGCCAAACTTATG | TTGTAGAACGTGATATCAGGAAAATAGC |
| *Smed-soxB-1* | transcription factor SOX-2 [Homo sapiens] | JX091078.1 | CACCAGCTTGGCAGAGAGATG | GCATAACCGGACGCCAGAT |
| *Smed-pbx-1* | pre-B-cell leukemia transcription factor 3 isoform 1 [Homo sapiens] | KC353351.1 | CCGCTCAAATAAATTCGGAAGAT | TCCAGTTACACCTTCAGCAATAAGC |
| *Smed-inx-13* | pannexin-2 isoform 1 [Homo sapiens] | JQ425145.1 | TTGTTCCAGGGCGTTTCG | CCTCTCCGGCAAAGATTGG |
| *Smed-smad6/7* | mothers against decapentaplegic homolog 7 isoform 4 [Homo sapiens] | JQ278719.1 | GACTGACGACAAACTGGAAATCG | CTTTAGCAGCATCCAGTGCAATT |
| *Smed-soxP-2* | transcription factor SOX-5 isoform c [Homo sapiens] | JQ425152.1 | CGATTGATATAATCTGTCATGCTGTTG | CACGCCCTGAATACGACATTG |
| *Smed-nlk-1* | serine/threonine-protein kinase NLK [Homo sapiens] | JQ425157.1 | CCCAAGAGGTTGTGACCCAATA | CCACATGTCGACAGCGAATG |
| *Smed-soxP-3* | transcription factor SOX-9 [Homo sapiens] | JQ425153.1 | CTGTCGATTGGCTGGAAACC | CCGGCCTCGATCAATTACAA |
| *Smed-egr-1* | E3 SUMO-protein ligase EGR2 isoform b [Homo sapiens] | JQ425156.1 | GCCGTGTCCGCTCATCAG | CGGCGAGAAGCCTTATTCCT |
| *Smed-zfp-1* | zinc finger protein 701 isoform 1 [Homo sapiens] | JQ425154.1 | GCTTCACTCAAAGATCAAATCTACAGA | TTAGAGCAAACTCCACATTTGTATGG |
| *Smed-fgfr-1* | fibroblast growth factor receptor 1 isoform 14 precursor [Homo sapiens] | JQ425160.1 | CATCCAATAAGTGCGCTTCTCTT | CAGTGGGAACTCCAGAATTGAAA |

**Table S2.** Selected gene groups for RT-PCR analysis of oxidative stress response genes.

| Gene name | Description | Schmidtea mediterranea genome database (SmedGD) | Primer F | Primer R |
|---|---|---|---|---|

| | | **Reference Sequence** | | |
|---|---|---|---|---|
| *Smed-gss* | glutathione synthetase [Homo sapiens] | SMU15015253 | TGCGCAGCAAACCGACTA | GCTATCGATGCATCCAAATCC |
| *Smed-gpx4* | glutathione peroxidase 4 (phospholipid hydroperoxidase) [Homo sapiens] | SMU15039906 | AAGGCGATGTTGTACTCGTTGTT | GAAGCTTATGCAACTCCTGCAA |
| *Smed-alh2* | mitochondrial aldehyde dehydrogenase 2 [Homo sapiens] | SMU15017380 | AGGACATCGACAAATCTTTGCA | GTTTGGGTGAATTGCTATGACGTA |
| *Smed-prx6* | peroxiredoxin-6 [Homo sapiens] | SMU15002827 | ATAGCTGACACGGATCGAGATCT | GGATCAAGGAAGTAAACAGCTCTTG |
| *Smed-txn* | thioredoxin [Homo sapiens] | SMU15025476 | TGGCTTCTTGTGATAAGTTAATTGTTG | CGAAAACGACGTCAGGAAATTC |
| *Smed-txnrd1* | thioredoxin reductase 1 [Homo sapiens] | SMU15008405 | GGTGGTCGGTGGTAGCTATGTAG | GAACCATAACGGTCGTATCACATC |
| *Smed-sod1* | superoxide dismutase [Cu-Zn] [Homo sapiens] | SMU15030255 | TGCACGAATGGCGAGATAATTA | TCCAATAACTCCACAAGCTACTCTTC |
| *Smed-pgd* | 6-phosphogluconate dehydrogenase, decarboxylating isoform 1 [Homo sapiens] | SMU15001176 | TTCACGCAAACTGTCAACATGTAT | GCATATCACCATTGCAATTGGA |
| *Smed-taldo* | TALDO1 protein [Homo sapiens] | SMU15040687 | CCAGGCCGTCGCATGT | GCCAATCATAAATTCTACCGACAA |
| *Smed-tkt* | transketolase isoform 1 [Homo sapiens] | SMU15039542 | CGATGAGGAGTTGCTGAGGAA | GGGAAATGATCCTCGACAGTGA |
| *Smed-fth* | FTH1 protein [Homo sapiens] | SMU15023437 | GAGAAACCAGCCAAAACTGATTG | TCTGTCAAGTGTGGATCAAAATGA |
| *Smed-gsts* | glutathione S-transferase omega-1 isoform 1 [Homo sapiens] | SMU15007045 | GAACCGAAAGTTGATCGAAGTAAAC | CATGTTCAATTCCATGGTGAGATAA |
| *Smed-ptk1* | protein kinase 1 [Homo sapiens] | SMU30003323 | CAATCAGTCCATGCCTGACTTG | GGTTGTGATAGTTCATAGTATGGTTTTGA |
| *Smed-gr2* | glutathione-disulfide reductase [Homo sapiens] | SMU15030732 | CGACAGAAAATCCCTGTAGCATT | ATTTGTTTGCTTCCCGATGAA |
| *Smed-ugt2b7* | UDP-glucuronosyltransferase 2B7 isoform 1 precursor [Homo sapiens] | SMU15001980 | TTTAATATTGAGCATTCCGATGGA | CAGACCTGTGAAATGTCAATAACGT |
| *Smed-g6pd* | glucose-6-phosphate 1-dehydrogenase isoform b [Homo sapiens] | SMU15012454 | AACCCGGTGAGGCGATTTAT | AACGTCATTGAGTCGCCAGTT |
| *Smed-cat* | catalase [Homo sapiens] | SMU15017976 | GGCTCATTTTGACCGAGAAAGA | GCACTCAAAGAAACCGAAAGCT |
| *Smed-3705* | kelch like ECH associated protein 1 [Homo sapiens] | SMU15003705 | AGATTATCCAGTGACACCTTTGCA | CCAGATTTTCCAGCGTCCAT |
| *Smed-9751* | kelch-like protein 20 [Homo sapiens] | SMU15039751 | TGATGTGCAGCAAAATGAGAAA | CCAGACAGAGACTTTGAAGGTGACT |
| *Smed-pgd* | 6-phosphogluconate dehydrogenase, decarboxylating isoform 1 [Homo sapiens] | SMU15001176 | TTCACGCAAACTGTCAACATGTAT | GCATATCACCATTGCAATTGGA |

| | | | | |
|---|---|---|---|---|
| *Smed-gcl* | glutamate--cysteine ligase catalytic subunit isoform a [Homo sapiens] | SMU15001870 | AATGGATCGAACCAGCATAATACTG | GACCATATGGATAGCCTGGAACTC |
| *Smed-bmp* | BMP8A protein [Homo sapiens] | SMU15037612 | CCATCAGAGAAAGTTCGCAGTAATTT | TGAGGCAATTTTTCCGACAAT |
| *Smed-alh1* | aldehyde dehydrogenase 1A1 [Homo sapiens] | SMU15001870 | GGAGTTTGTTCAGCGGGTTTAA | TTCATGCTGGCTGTTTTCAAATT |

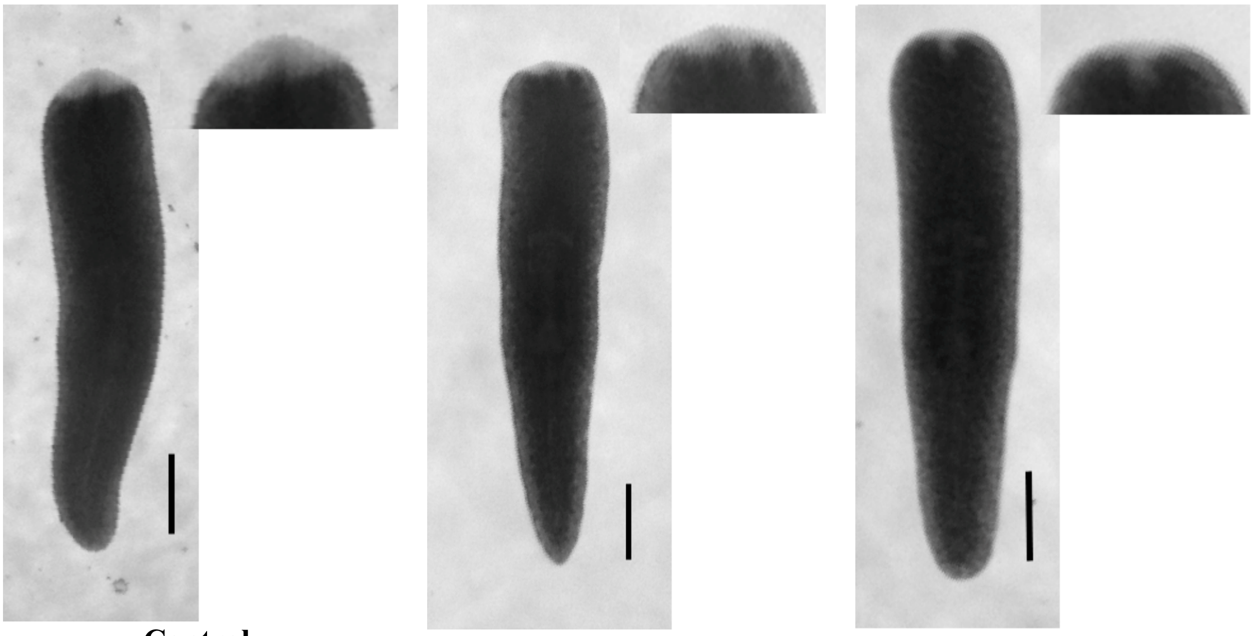

**Control** **10 Gy** **15 Gy**

**Figure S1**. The representative photographs of regenerating planarians for blasthema growth rate analysis by means of computer morphometry. Depicted are planarians on third day after decapitation, the control and planarians after X-ray irradiation with doses of 10 and 15 Gy. Bar – 1 mm

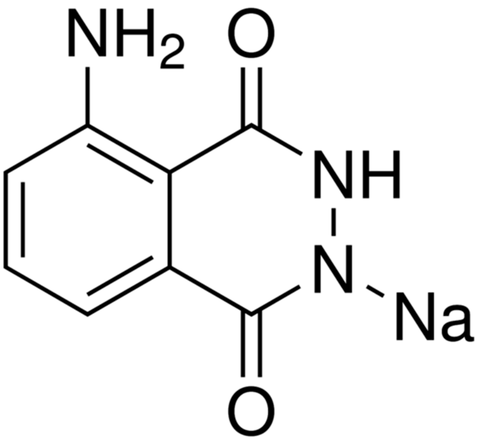


**Figure S2**. Structural chemical formula of Tameron (5-amino-2,3-dihydro-1,4-phthalazinedione sodium salt).

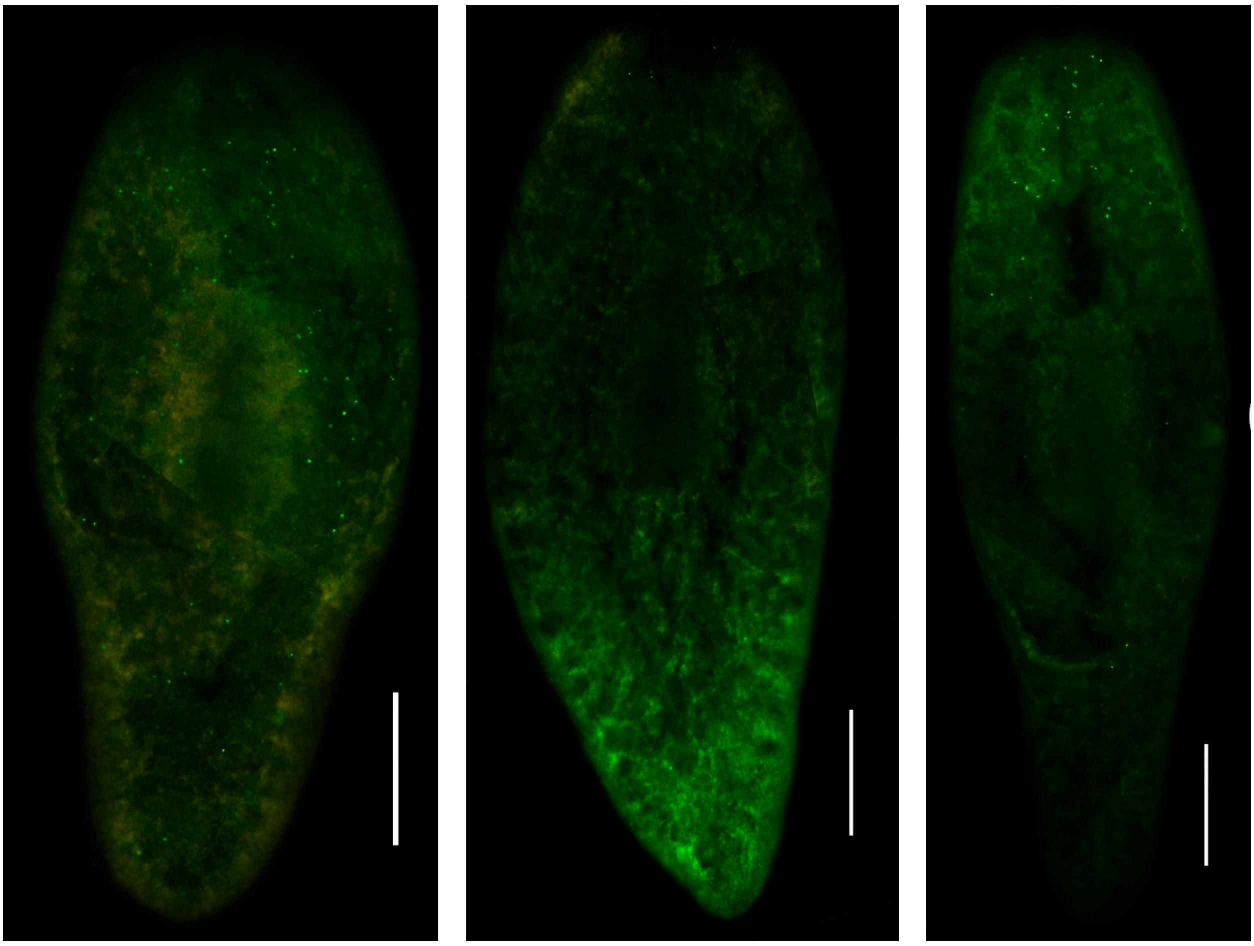

**Control** **15 Gy** **15 Gy+Tameron**

**Figure S3**. Mitotic cells in planarian bodies (whole-mount Immunocytochemistry) 7 days after 15 Gy irradiation. Bar – 1 mm
